# Prevalence of Suicide Attempts among College Students in China: A Meta-Analysis

**DOI:** 10.1371/journal.pone.0116303

**Published:** 2015-02-09

**Authors:** Lin-Sheng Yang, Zhi-Hua Zhang, Liang Sun, Ye-Huan Sun, Dong-Qing Ye

**Affiliations:** 1 Department of Epidemiology and Biostatistics, School of Public Health, Anhui Medical University, Hefei, China; 2 Department of Public Health, Fuyang Center for Disease Control and Prevention, Fuyang, China; Chiba University Center for Forensic Mental Health, Japan

## Abstract

**Background:**

Suicide is the leading cause of death among 15–34 year olds in China, but no national data are available on the suicide and suicide attempts rates of college students, a sub-group of youth with 23 million. Several studies have reported the prevalence of suicide attempts among college students, however, no meta-analysis pooling the prevalence of suicide attempts is found.

**Objective and Methods:**

This study aims to estimate the pooled prevalence of suicide attempts among college students in China. The relevant studies up to August 2014 were systematically searched via electronic databases (PubMed-Medline, Embase, Chinese Wanfang database, Chinese National Knowledge Infrastructure and Chinese VIP database). We only selected original articles that either reported the prevalence of suicide attempts or sufficient data for calculating the prevalence.

**Results:**

A total of 29 eligible studies, with 88,225 college students, were finally included. The maximum and minimum reported prevalences of suicide attempts among college students in China were 0.4% and 10.5%, respectively. The pooled prevalence of suicide attempts was 2.8% (95%*CI*: 2.3%–3.3%). Subgroup analyses showed that the pooled estimate of prevalence of life time suicide attempts was 2.7% (95%*CI*: 2.1%–3.3%), and 12-month suicide attempts was 2.9% (95%*CI*: 2.0%–3.8%). The prevalence for males was 2.4% (95%*CI*: 1.8%–3.0%), and for females was 2.7% (95%*CI*: 1.9%–3.7%). The prevalences among college students in grade 1 through 4 were 2.8% (95%*CI*: 1.7%–3.8%), 1.8% (95%*CI*: 1.2%–2.3%), 2.0% (95%*CI*: 0.8%–3.1%), and 2.9% (95%*CI*: 0.1%–6.7%), respectively. The prevalences among college students from rural and urban areas were 5.1% (95%*CI*: 2.8%–7.5%) and 3.7% (95%*CI*: 1.4%–5.9%), respectively.

**Conclusions:**

2.8% prevalence of suicide attempts and more than 600,000 suicide attempters among college students indicate that suicide attempt among college students is an important public health problem in China. More attention should be paid to the current situation.

## Introduction

Suicide is the leading cause of death among 15–34 year olds in China, accounting for 19% of all deaths [1]. Although the overall suicide rate in China decreases significantly over the past decade, rates in young people 15–24 years of age do not reduce [2–3]. It is suggested that more attention should be paid to this population. Nevertheless, the youth is a heterogeneous population at risk for suicide and little is known about rates of sub-groups of the youth except that by sex and age groups because of limits of the Chinese vital registration system [4], for example, no national data are available on the suicide rate of college students, although there are more than 23 million college students in China [5] and a considerable number of suicide cases among this population have been reported. As a result, more data on the prevalence of suicidal ideation and attempts among college students are needed, according to the assumption that such data would be useful for understanding completed suicides. Although suicide attempt need not result in death [6], it is a significant predictor of subsequent completed suicide, as well as important in its own right as an indicator of extreme psychological distress [7].

Current estimates of the prevalence of suicide attempts among college students in China are almost drawn from school-based cross-sectional investigations. These investigations provided valuable information, but they were limited because most of them focused on one or several universities in one province rather than nationally representative sample of this population. There has been no meta-analysis pooling the prevalence of suicide attempts across different provinces to date. The present study therefore aims to estimate the overall pooled prevalence of suicide attempts among college students in China. We also estimate the pooled prevalences of suicide attempts in different subgroups of college students.

## Methods

### Search strategy

The relevant studies were searched via six electronic databases: Medline, Embase, ISI Web of Science, Chinese WanFang Database, Chinese National Knowledge Infrastructure (CNKI) and Chinese VIP database. Data searches were carried out on 20 August 2014, without restrictions regarding publication year. Search terms were initially on the basis of words used in the article titles, abstracts, subheadings and keywords, then tested in a pilot and refined afterwards. The following search terms were used in the final search:

#1 TS = (suicide or suicide attempt or suicidal behavior or suicidal ideation)
#2 TS = (“college student” or “university student” or “undergraduate” or “medical student”)
#3 TS = (China or Chinese or Hongkong or Macao or Taiwan)
#4 #1 and #2 and #3

To supplement the electronic searches, we also conducted searches for the reference lists of relevant articles.

### Eligibility criterias

Studies were included in the review if they met the following criterias: (1) The study either reported the prevalence of suicide attempts or sufficient data for calculating the prevalence; (2) The study was conducted in China; (3) If the studies were based on the same sample, only the study with greatest epidemiological quality was selected; (4) Articles were written in English or Chinese.

### Study selection and data abstraction

After initial evaluation, two reviewers independently and carefully reviewed the articles and filled out a standard quality assessment checklist with 11 questions concerning the methodological aspects of cross-sectional studies for each study [8].

The following data were extracted for each study: authors of study, years published, province, study design, total number of subjects recruited, and number of suicide attempters. We also extracted the number of suicide attempters according to gender, grade, and Urban/Rural, in order to estimate the prevalence of suicide attempts in sub-groups.

### Quality assessment

Agency for Healthcare Research and Quality (AHRQ) was used to assess the quality of cross-sectional studies [8]. AHRQ was an 11-item instrument with a yes/no/unclear response option: the “Yes” would be scored “1”, “No” or “unclear” was scored “0”. Articles were scored as follows: 0–3 = low quality; 4–7 = moderate quality; 8–11 = high quality.

### Statistical analysis

We first transformed prevalences via the Freeman-Tukey double arcsine method [9] then performed an inverse-variance weighted. The transformed prevalences are weighted very slightly towards 50% and studies with prevalences of zero can thus be included in the analysis. The pooled prevalences were calculated as the back-transform of the weighted mean of the transformed prevalences [10].

All statistical analyses were done using STATA 10 (Stata Corporation, College Station, Texas, USA). We used Cochran *Q* and the *I^2^* statistic [11] to explore the variation between studies and found significant heterogeneity between the study findings. So, random effect model was used to estimate the pooled prevalence and 95% *CIs*. In order to explore the potential heterogeneity between studies and the prevalences of suicide attempts with different characters such as gender, grade, et al., we also conducted subgroups analysis. A funnel plot (prevalence versus standard error) was used to explore the publication bias [12]. Funnel-plot asymmetry was further assessed by the method of Begg’ test and the modified Egger’s linear regression test.

## Results

### Search results

**Figure 1:**
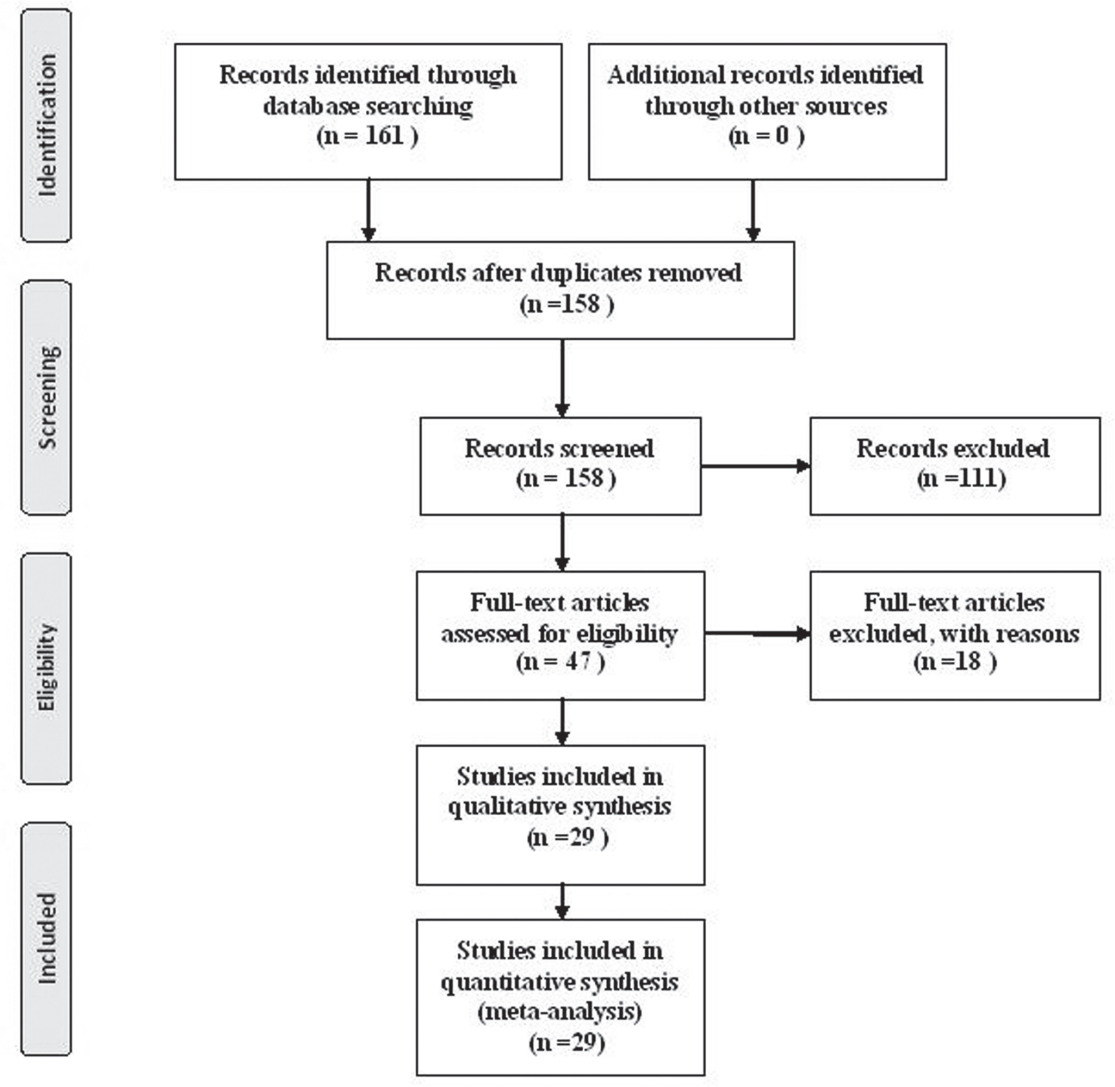
Flow diagram of assessment of studies identified in the meta-analysis.

The electronic database searches initially yielded 161 papers (41 papers in English and 120 in Chinese). Of these, 129 were subsequently removed due to either duplication or a failure to meet the inclusion criteria. The remaining 47 papers were retrieved for full-text screening. In the end, 29 papers were entered into this meta-analysis. See Fig. 1.

### Characteristics of included studies

Five of the studies were published in English and 24 in Chinese.16 studies reported the life time prevalence of suicide attempts and 13 studies reported the 12-month prevalence. Studies were conducted in mainland China (Anhui [20, 22, 30, 35–36, 40–41], Guangdong [25, 37–38], Hunan [18, 28], Ningxia [23, 34], Shanghai [39], Heilongjiang [14], Hubei [13], Sichuan [17], Shanxi [19], Chongqing [24], Jilin [26], Yunnan [27], Henan [31], Shangdong [32], and sample of several provinces [15, 16, 33]), and Taiwan [21, 29]. 88,225 college students were finally included.

### Quality assessment

Four studies were of high quality, other studies were of moderate quality (see Table 1).

**Table 1:** Key features of the included studies.

**References**	**Citation**	**Location**	**Number of school**	**Period**	**Sample size**			**Prevalence (95%*CI*) (%)**	**Quality Score**
					**overall**	**Male**	**female**		
13	ZQ You et al.(2014)	Hubei	6	life	6096	3203	2785	1.9(1.6–2.2)	8
14	L Wang et al.(2014)	Heilongjiang	6	Life	5240	2563	2682	1.0(0.7–1.3)	8
15	JB Zhao et al. (2013)	Six provinces	12	Life	8202	3094	5108	3.0(2.6–3.4)	8
16	JB Zhao et al. (2013)	Sampled from 1949 schools	10	Life	1168	542	626	1.9(1.1–2.7)	8
17	D Liu et al. (2013)	Sichuan	1	Life	1371	691	680	1.4(0.8–2.0)	5
18	YL Deng et al.(2012)	Hunan	6	Life	2166	859	1306	4.3(3.4–5.2)	5
19	RR Chen et al. (2011)	Shanxi	1	Life	1055	519	635	6.2(4.7–7.7)	4
20	X xin et al. (2010)	Anhui	2	Life	800	390	410	5.5(3.9–7.1)	4
21	SS Gau et al.(2009)	Taiwan	1	Life	2918	1416	1503	1.2(0.8–1.6)	6
22	HY Cao et al.(2009)	Anhui	3	Life	10344	4780	5564	1.4(1.2–1.6)	7
23	YX Shang et al.(2008)	Ningxia	1	Life	1484	505	979	5.2(4.1–6.3)	5
24	L Kuang et al.(2008)	Chongqing	11	Life	9808	5381	4427	1.7(1.4–2.0)	7
25	SS Fen et al.(2008)	Guangdong	8	Life	1863	920	943	3.4(2.6–4.2)	6
26	YF Cheng et al.(2008)	Jilin	1	Life	1822	1223	599	0.4(0.1–0.7)	6
27	QQ Liu et al.(2007)	Yunnan	13	Life	3313	1671	1640	4.6(3.9–5.3)	6
28	HL XU et al.(2004)	Hunan	1	Life	610	-	-	3.0(1.6–4.4)	5
29	CH Chou et al.(2013)	Taiwan	1	12 mth	2835	1263	1572	10.5(9.4–11.6)	6
30	YH Wan et al.(2012)	Anhui	1	12 mth	4063	1895	2168	0.6(5.2–6.6)	7
31	AH Ma et al. (2010)	Henan	5	12 mth	1285	544	741	1.2(0.6–1.8)	5
32	LH Li et al.(2010)	Shangdong	1	12 mth	592	279	313	4.7(3.0–6.4)	4
33	R Gao et al.(2010)	data from eight cities in China	8	12 mth	5152	-	-	1.0(0.7–1.3)	7
34	YX Shan et al.(2008)	Ningxia	2	12 mth	2678	958	1720	5.5(4.6–6.4)	6
35	YG Fan et al.(2008)	Anhui	3	12 mth	3517	1882	1635	1.5(1.1–1.9)	6
36	YG Fan et al.(2008)	Anhui	1	12 mth	2160	1198	962	0.4(0.1–0.7)	6
37	WJ Shi et al.(2007)	Guangdong	8	12 mth	2564	1278	1286	6.0(5.1–6.9)	6
38	LN Zen et al.(2006)	Guangdong	2	12 mth	1245	792	453	2.5(1.6–3.4)	6
39	JP Zhu et al.(2006)	Shanghai	7	12 mth	1722	890	832	1.2(0.7–1.7)	5
40	R Zhang et al.(2002)	Anhui	5	12 mth	1267	903	364	2.0(1.2–2.8)	5
41	FB Tao et al.(1999)	Anhui	4	12 mth	884	636	248	1.9(1.0–2.8)	5

### Overall prevalence of suicidal attempt

**Figure 2:**
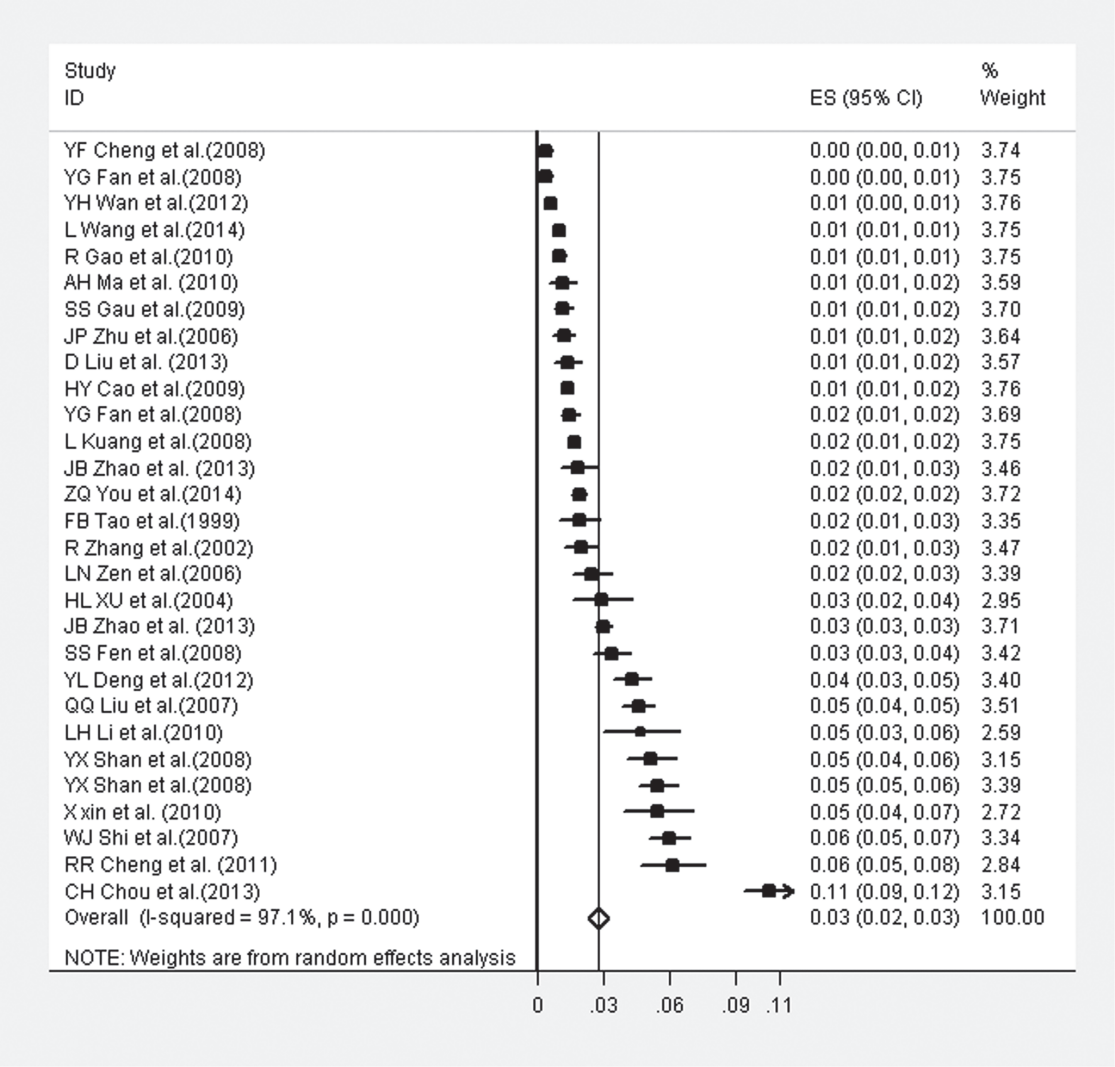
Forest plot of the 29 studies included in the meta-analysis.

Suicide attempts prevalences varied from 0.4% to 10.5% and were displayed as forest plots in Fig. 2. The heterogeneity of the studies was high (*Q* = 957.71, *P*<0.001; *I^2^* = 97.1%). The pooled prevalence estimate for suicide attempts via random effect model was 2.8% (95%*CI*: 2.3%–3.3%).

### Publication bias

**Figure 3:**
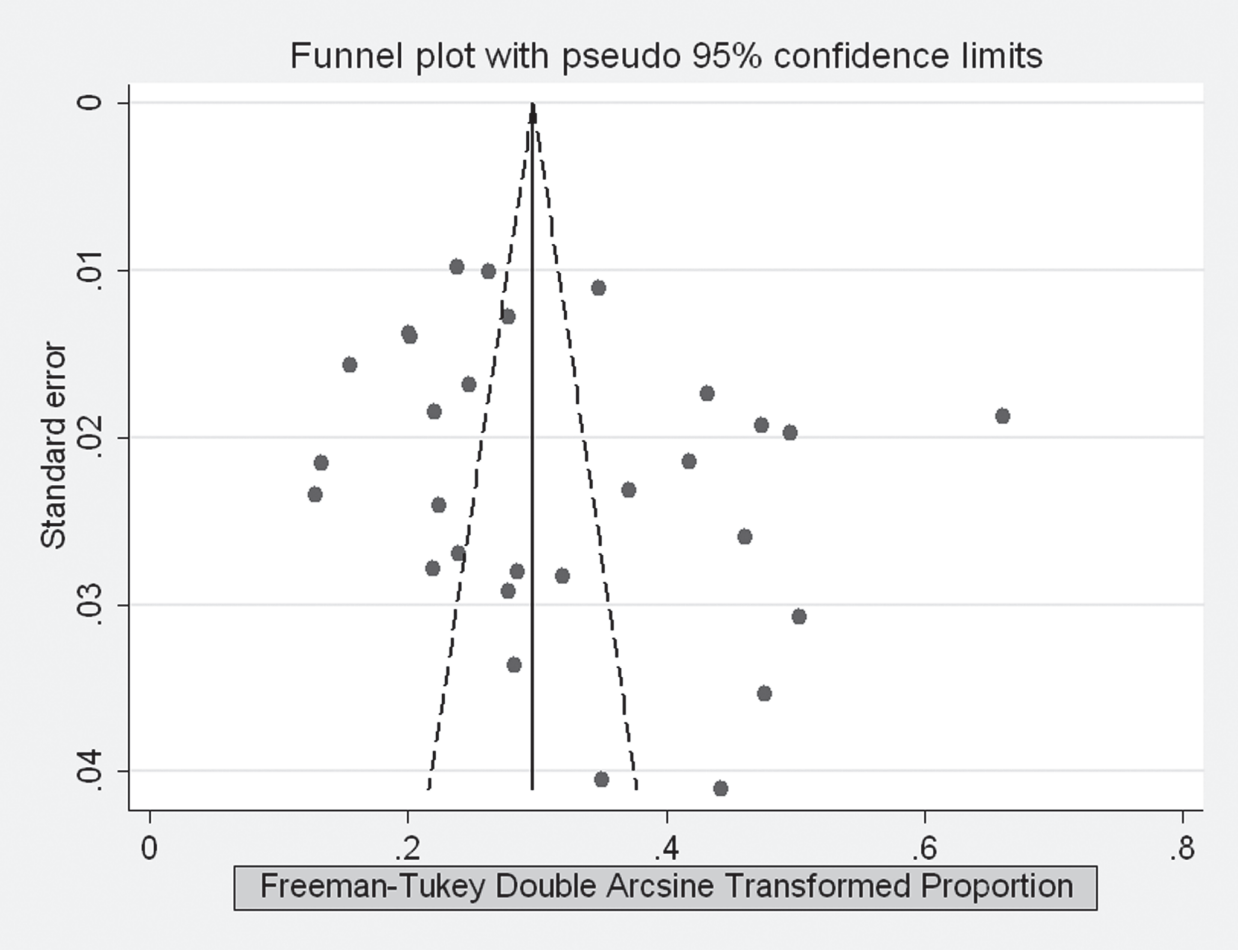
Funnel plot of the 29 studies included in the meta-analysis.

Both Begg’ test (*Z* = 1.68, *P* = 0.094) and Egg’s test (*t* = 1.44, *P* = 0.161) showed no potential risk of publication bias, although the funnel plot was slightly asymmetrical (see Fig. 3).

### Subgroup analysis

The results of subgroup analysis are shown in Table 2. Of the 29 included studies, 16 studies reported the life time prevalence of suicide attempts and 13 studies reported the 12-month prevalence. The pooled prevalence of life time suicide attempts was 2.7% (*95%CI*: 2.1%–3.3%), and 12-month suicide attempts was 2.9% (*95%CI*: 2.0%–3.8%). The suicide attempts prevalence was reported separately for males and females in 14 studies. The pooled prevalence for males was 2.4% (95%*CI*: 1.8%–3.0%), and for the females was 2.7% (95%*CI*: 1.9%–3.7%). Nine studies independently reported the prevalence among college students in grade one, 6 studies in grade two, 4 studies in grade three, and 2 studies in grade four. The pooled prevalence in grade one was 2.8% (95%*CI*: 1.7%–3.8%), 1.8% (95%*CI*: 1.2%–2.3%) in grade two, 2.0% (95%*CI*: 0.8%–3.1%) in grade three, and 2.9% (95%*CI*: 0.1%–6.7%) in grade four. Additionally, three studies reported prevalences among students from urban and rural areas. The pooled prevalences were 3.7% (95%*CI*: 1.4%–5.9%) and 5.1% (95%*CI*: 2.8%–7.5%), respectively.

**Table 2:** The prevalence of suicide attempt in different subgroup of college students in China.

**Subgroup**	**No. studies**	***P*(%)**	***95%CI* for *P***	**Heterogeneity**			**Begg’s test**		**Egg’s test**	
				***χ*^2^**	***P***	***I*^2^(%)**	***Z***	***P***	***t***	***P***
Period										
Life time	16	2.7	2.1–3.3	380.22	<0.001	96.1	1.13	0.26	1.63	0.13
12-month	13	2.9	2.0–3.8	292.49	<0.001	96.2	1.46	0.16	0.5	0.63
Gender										
Male	14	2.4	1.8–3.0	281	<0.001	94.7	1.53	0.12	1.93	0.08
Female	14	2.7	1.9–3.7	337	<0.001	95.9	1.00	0.32	0.28	0.79
Grade										
1	9	2.8	1.7–3.8	68.99	<0.001	88.2	0.6	0.55	1.7	0.15
2	6	1.8	1.2–2.3	43.84	<0.001	82.9	1.2	0.16	1.6	0.13
3	4	2.5	0.8–4.3	19.07	<0.001	84.3	-0.34	0.99	-0.59	0.61
4	2	2.9	0.1–6.7	5.70	0.017	82.5	<0.001	>0.99	-	-
Urban/Rural										
Urban	3	3.7	1.4–5.9	21.56	<0.001	90.7	1.04	0.30	-1.53	0.37
Rural	3	5.1	2.8–7.5	18.86	<0.001	89.4	1.04	0.30	-2.00	0.30

## Discussion

To our knowledge, this study is the first meta-analysis pooling the prevalence of suicide attempts among college students in China. In the current meta-analysis, 29 eligible studies, with a total of 88,225 subjects, were included. We found that, among college students in China, the prevalence of suicide attempts ranged from 0.4% to 10.5%, and the pooled prevalence was 2.8% (95%*CI*: 2.3%–3.3%). This result suggests more than 600,000 college students have suicide attempts in China.

The 2.8% prevalence of suicide attempts is well above reports for the general population in China, such as the 1.0% prevalence reported by Ma X et al. [42], Lee S et al. [43], Wang Z et al. [44] and the WHO World Mental Health surveys [45], respectively. An earlier report for Chinese sub-sample (mean age = 43) by the WHO SUPRE-MISS study was 2.4%, slightly less than our estimate [46]. Although the variations in prevalences are probably due to sample selection (e.g., None of nationally representative study of the general population is found) and variability in the methods used to assess suicidal behaviors, such consistent results suggest that the risk for suicide attempts among college students is higher than the general population in China, which is different from reports in west. For example, the data from American College Health Association in 2008 and 2010 showed 1.3% [47] and 1.2% [48] prevalence among college students, respectively, both less than the report for US adults (1.9%–8.7%; IQR, 3.0%–5.1%) [49].

However, 2.8% lifetime prevalence of suicide attempts among college students is less than reports for adolescents in China. For instance, a school-based survey with 13,817 middle school students found 4.7% prevalence [50]. A sample consisted of 2,579 Grade 8 students from 28 secondary schools in Hong Kong also found nearly 4.0% of adolescents attempted suicide in the preceding 12 months [51]. Another survey with 2013 Chinese students (including about 400 college students) showed 3.5% prevalence of suicide attempts [52].

Compared with reports for college students from other countries, our estimate is above the reports from American College Health Association (1.2%–1.3%) [47–48], but less than the reports in India (4.0%) [53], Indonesian (7.9%) [54], and Turkish (7.1%) [55].

### Life time/12 months

Contrary to our expectations, 2.7% life time prevalence of suicide attempts was less than 2.9% 12-month prevalence. This unexpected result is related to the highest 12-month prevalence (10.5%) [29]. If the highest prevalence is excluded, the pooled 12-month prevalence is 2.2% (95%CI: 1.5%–2.9%), less than life time prevalence. But even so, the difference between life time prevalence and 12-month prevalence may be underestimated because of recall bias.

### Gender

Of 14 studies reporting prevalence on males and female, eight studies showed that the prevalence of suicide attempts was higher in females than males, other 6 studies exhibited opposite results. As expected, the pooled prevalence of suicide attempts was higher in females than in males (2.7% vs.2.4%), however, the difference was not significant (Z = 0.95, P = 0.344). Clear, the 1:1 male-to-female prevalence ratio among college students is different from general population in China [42–43] and in western countries [49]. However, similar results can be also found among college students in other countries [48, 53, 55], In Turkey, prevalences in male and female students were 6.8% and 6.7%, respectively [55]. These results may represent a fact that there are no gender difference at risk for suicide attempts among college students, which is different from adolescents and adults. A recent longitudinal study [56] conducted among European American adolescents indirectly supports this speculation. The yearly prevalence of suicide attempts in female was higher than males across the ages, and both increased through mid-adolescence and then declined. But the prevalence for attempt peaked at age 16 in females, earlier than males (at age 17), and declined more rapidly in females than males. Such results indicate that the difference of suicide attempts prevalence between females and males should be gradually reduced, or even disappeared at a particular older age group, although this speculation has the risk of errors because only adolescents ages 11–19 were included in this study.

### Grade

With respect to grade difference, we found the distribution of prevalences among students grade 1 through 4 is “U”, with higher prevalences in grade 1 and 4 than in grade 2 and 3. This phenomenon could be explained that students in grade 1 and 4 faced more stress. Students in grade 1 may face significant and more stresses associated with adjusting to a new social environment, increased academic demands, physical separation from parents, and alterations in social support networks [57–58]. While great employment pressure will follow the students in grade 4 [59]. A survey among college students by Guo SF [60] showed that employment pressure was considered the biggest source of stress by 51.4% of participants.

### Urban/Rural

The huge difference of suicidal behaviors between urban and rural areas was an important characteristic in China [1–2]. As expected, this study found a higher risk for suicide attempts in college students from rural areas than from urban areas. The relatively higher prevalence college students from rural areas may be related to more exposure to risk factors for suicide, such as more possibility to witness peers’ or adults’ suicide, lower education of parents and family income, et al.

### Limitations

Our estimates about prevalences of suicide attempts among Chinese college students should be interpreted with caution because of the great heterogeneity between studies. Although we performed subgroup analyses by life time/12 months, gender, grade, and urban/rural, and found that life time/12 months, grade, and urban/rural may be the sources of between-study heterogeneity, the factors affecting suicide attempt have so many, not being addressed in this meta-analysis. First, no unique method used to assess suicidal behaviors was found in studies, which may be a source of high heterogeneity between studies. However, we could not extract sufficient data to assess it. Second, the difference of students’ major may play a role in between-study heterogeneity [35, 37], but it was also not included in the current study because most studies either did not collect or report differences according to students’ major. Third, of 34 provinces in China, only 15 provinces reported the prevalence of suicide attempts among college students, which indicated the sample’s representation was not enough.

## Conclusions

2.8% prevalence of suicide attempts and more than 600,000 suicide attempters among college students indicate that suicide attempt among college students is an important public health problem in China. Our results also suggest that college students may have their own suicidal behavior characters, different from that of general population.

More suicide prevention efforts should be done to prevent suicide attempt among college students, especially the students in grade 1, 4 and from rural areas given that they are at more risk for suicide attempts than others. For instance, university psychologists should pay more attention to this sub-group of students, including talking with them about the risk for suicide, providing interventions for those at imminent risk for suicidal behavior, and referring patients for expert assessment and treatment.

### Ethical Standard

This review did not involve animal or human experimentation.

## Supporting Information

S1 PRISMA Checklist PRISMA 2009 Checklist. (DOCX)
